# Special Issue “Genetic Regulation of Plant Growth and Protection”

**DOI:** 10.3390/ijms262311535

**Published:** 2025-11-28

**Authors:** Jia Li, Xiaohong Tong, Zhiyong Li, Yuxuan Hou

**Affiliations:** State Key Laboratory of Rice Biology and Breeding, China National Rice Research Institute, Hangzhou 311400, China; 2022011010015@stu.hznu.cn (J.L.); tongxiaohong@caas.cn (X.T.)

Plants in natural environments are subject to diverse biotic and abiotic stresses, such as drought, low temperatures, and pests, which significantly impact their growth, development, and yield. To address these challenges, plants have evolved complex genetic regulatory networks that precisely control gene expression to balance growth and stress responses. In recent years, advancements in molecular biology and genomics have progressively unraveled the genetic mechanisms underlying plant growth and defense, including the roles of transcription factors, epigenetic modifications, hormone signaling pathways, and non-coding RNAs. The emergence of gene-editing technologies such as CRISPR-Cas9 has provided revolutionary tools for precisely modifying these regulatory genes and breeding stress-resistant crops. In this review, we summarize recent progress in the genetic regulation of plant growth and defense, focusing on key genes and their molecular mechanisms reported in *IJMS* (*International Journal of Molecular Sciences*), and discuss their potential applications in sustainable agriculture.

## 1. Genetic Regulation of Plant Growth and Development

In recent years, research in the field of plant functional genomics has progressively shifted from characterizing genes controlling individual traits to elucidating the complex regulatory networks underlying the coordinated development of multiple traits. The discovery and functional validation of key genes—ranging from transcription factors regulating abscission layer formation to phytohormone biosynthesis genes conserved across species and organelle-specific factors essential for photosynthetic efficiency—collectively delineate a multi-layered regulatory blueprint that governs plant development from organelle function to organ morphology, and from vegetative growth to reproductive success.

Xie et al. identified that in the BELL transcription factor family of *Zizania latifolia*, the genes *ZlqSH1a* and *ZlqSH1b* are homologs of the key shattering gene in rice. Their overexpression in rice promotes complete abscission zone development by regulating hormone signaling and cell wall metabolism-related genes (upregulation of *ERF* and downregulation of *PG1/PG2*), elucidating the genetic basis of strong seed shattering in *Zizania latifolia* and providing target genes for the domestication of varieties with moderate shattering (contribution 1). Liu et al. constructed a high spatiotemporal resolution multi-omics atlas of wheat spike development, revealing that cytokinin treatment significantly reduces basal spikelet abortion rates and increases fertile spikelet numbers, while abscisic acid inhibits overall spike development [1]. Regarding plant growth and biomass enhancement, the heterologous overexpression of the rice brassinosteroid (BR) biosynthesis key gene *OsCYP85A1* in poplar activates growth signaling pathways and secondary cell wall synthesis genes, significantly increasing plant height, stem diameter, and root system development, while also enhancing carbon assimilation capacity and disease resistance. These findings validate the cross-species conservation of the gene’s growth-promoting function (contribution 2). Liu et al. employed interdisciplinary techniques including 3D imaging, genome-wide association study (GWAS), single-cell transcriptomics, and molecular biology to identify 72 key genes influencing root architecture for the first time. They further elucidated that the *PsiSKP2B* gene positively regulates poplar lateral root development by ubiquitin-mediated degradation of PsiZHD9 and PsiWOX4 proteins, thereby increasing root auxin content, providing a key genetic target for forest tree root improvement [2]. Rice lipid synthesis and regulatory genes are involved not only in fundamental processes such as membrane construction and energy storage but also influence plant fertility, nutritional quality, and stress resistance by regulating anther cuticle formation, grain composition, and wax accumulation (contribution 3). The *OsRNE* gene identified by Fang et al. ensures normal chloroplast development and photosynthesis by regulating chloroplast RNA metabolism, making it critical for rice seedling survival and growth (contribution 4). 

These findings collectively outline a multi-layered regulatory network governing plant development, from organelle biogenesis to organ formation, and from vegetative growth to reproductive success, providing critical theoretical foundations and genetic resources for crop genetic improvement. Further elucidation of the interactions among these genes and their environmental response mechanisms will help reveal the coordinated regulatory principles underlying plant growth, development, and stress adaptation. By integrating multi-omics data mining with gene-editing technologies, the precise modification of functional combinations of key hub genes is expected to enable synergistic improvements in crop yield, quality, and stress resilience, thereby advancing the development of next-generation intelligent design breeding systems.

## 2. Genetic Regulation of Plant Stress Tolerance

During growth and development, plants inevitably face various abiotic stresses, such as drought, low temperature, salinity, and nutrient deficiency. A profound understanding of the molecular regulatory networks underlying plant responses to these adversities is crucial for genetic improvement of stress resistance in crops. The authors of recent studies across multiple species have achieved breakthrough advancements in this field.

In tobacco, *NtAITRs* function as transcriptional repressors in the ABA signaling pathway, negatively regulating drought tolerance. Gene editing-mediated knockout of this gene family significantly enhanced drought resistance without impairing normal growth (contribution 5). Previously, Yue et al. demonstrated that heterologous expression of the potato *NAC1* gene in *Arabidopsis* not only improved seed germination rates and green leaf retention but also reduced ROS accumulation while simultaneously increasing proline levels, thereby enhancing salt tolerance [3]. In rice, the “SAPK10-ABF1-*TPS2*” pathway elucidates a novel mechanism whereby the ABA signaling component OsABF1, upon phosphorylation and activation by SAPK10, directly promotes the expression of the trehalose biosynthesis gene *TPS2* to enhance cold tolerance (contribution 6). Another bZIP transcription factor, *OsbZIP72*, positively regulates cold tolerance at both seedling and booting stages. Knockout mutants exhibited significantly reduced seedling survival rates under 4 °C stress, whereas overexpression lines showed improved survival and higher seed-setting rates under low-temperature conditions during booting [4]. Furthermore, *OsbZIP72* promotes jasmonic acid biosynthesis via the “SAPK10-bZIP72-*AOC*” pathway while concurrently suppressing seed germination [5]. In oil-tea camellia, the SPX family gene *CoSPX-MFS3* acts as a key positive regulator of plant adaptation to low-phosphorus conditions by modulating organic acid secretion, phosphate transporter gene expression, and vacuolar phosphate homeostasis (contribution 7). Fang et al. revealed the molecular mechanism of phosphate anion efflux mediated by *Arabidopsis PHO1;H1* and its regulation by inositol polyphosphates. Both *Arabidopsis* PHO1 and its homolog PHO1;H1 are typical SPX-EXS family phosphate transporters involved in loading inorganic phosphate into the xylem vessel for root-to-shoot translocation [6]. Collectively, these findings substantially advance our understanding of plant adaptation mechanisms to drought, cold, and nutrient deficiencies at multiple levels, providing diverse genetic targets and strategic approaches for the molecular breeding of stress-resilient crops.

In summary, the discovery and functional characterization of key genes—from transcription factors in tobacco and rice (*NtAITRs*, *OsABF1*, and *OsbZIP72*) to transporter proteins in oil-tea camellia and Arabidopsis (*CoSPX-MFS3* and *PHO1;H1*)—systematically reveal multi-layered strategies employed by plants to combat environmental stresses through integrated hormone signaling, reprogrammed metabolic pathways, and optimized nutrient transport. These advances not only deepen our understanding of plant stress adaptation mechanisms but also mark a transition in crop breeding from traditional phenotype-based selection toward precision molecular design breeding.

## 3. Genetic Regulation of Plant Biotic Stress Defense

The authors of recent studies have multilaterally elucidated the complex dynamics of plant–pathogen interactions, spanning from pathogenic virulence strategies to plant immune networks. On the pathogen side, the novel rice bacterial blight strain LA20 utilizes its unique repertoire of TAL effectors (*Tal1b* and *Tal4*) to precisely target rice susceptibility genes *OsSWEET1*1 and *OsTFX1*, thereby overcoming resistance mediated by major R genes including *xa5*, *xa13*, and *Xa7* (contribution 8). This finding reveals how pathogen structural variation in effectors enables breakthrough of host resistance barriers, informing new strategies for developing broad-spectrum resistant cultivars. On the plant side, multiple key regulatory genes enhance resistance through distinct pathways. In tomato, *SIWRKY80* functions as a positive regulatory transcription factor that establishes a multi-layered defense network against root-knot nematodes by coordinately activating salicylic acid, jasmonic acid, and ethylene signaling pathways, maintaining ROS metabolic homeostasis, and enhancing root vitality (contribution 9). Furthermore, *SIWRKY80* participates in MeJA response and positively regulates salt-alkali stress tolerance. The SIWRKY80 protein directly binds to promoters of *SISPDS2* and *SINHX4*, activating their transcription to promote spermidine synthesis and Na+/K+ homeostasis, providing new theoretical foundations for understanding MeJA-mediated stress resistance and tomato production. In rice, *OsSPL42* acts as a putative negative regulator whose loss-of-function leads to ROS accumulation, programmed cell death, and lignin deposition, consequently enhancing resistance to specific *Xoo* strains [7]. Simultaneously, OsSPL42 interacts with multiple organellar RNA editing factors (*OsMORF8-1*/*OsMORF8-2*), affecting RNA editing processes (contribution 10). The *spl42* mutant exhibits impaired chloroplast development with abnormal thylakoid morphology, accumulated O^2−^ and H_2_O_2_, and enhanced activities of ROS-scavenging enzymes (CATB, AOX1a, and AOX1b). Transcriptional analysis results showed significant downregulation of chloroplast development and chlorophyll biosynthesis genes; in comparison, senescence-associated, ROS-producing, and defense-related genes were upregulated [8]. In potato, *StPAM16-1* was identified as a negative immune regulator that suppresses the response to common scab toxin through interaction with *COP9* signalosome subunit *StCSN5*. It has been demonstrated that silencing this gene enhances disease resistance (contribution 11).

These findings, spanning from pathogen virulence evolution to multi-layered plant immune regulation, provide crucial targets and theoretical foundations for designing durable and broad-spectrum disease resistance breeding strategies. The authors of future studies should focus on integrating multi-omics data to systematically elucidate key nodes within disease resistance signaling networks and their dynamic regulatory mechanisms, while simultaneously developing novel gene editing-based breeding technologies that enhance crop resistance without compromising agricultural product quality and safety. Through cross-species functional validation of candidate genes, we can accelerate the exploration and utilization of disease resistance genetic resources, thereby establishing scientific and technological support for building sustainable plant disease management systems.

## 4. Conclusions and Perspectives

In this review, we summarize the genetic regulatory mechanisms underlying plant growth and defense, covering the latest research advances in three major aspects: plant growth and development, abiotic stress tolerance, and biotic stress resistance. Through molecular biology and genomics technologies, scientists have revealed the crucial roles of multiple key genes and their regulatory networks in plant responses to environmental stresses and maintenance of growth and development. Researchers have found that BELL transcription factor family members (such as *ZlqSH1a* and *ZlqSH1b*) play a pivotal role in seed abscission by regulating phytohormone signaling and cell wall metabolic pathways to influence abscission layer development. Overexpression of the brassinosteroid (BR) biosynthesis gene *OsCYP85A1* significantly promotes plant growth while concurrently enhancing xylem formation and disease resistance. Lipid biosynthesis-related genes (*KCS* and *OsFBN1*) play multiple roles in plant growth and development, stress responses, and quality formation. The chloroplast development gene *OsRNE* affects photosynthesis by regulating chloroplast RNA metabolism, which is crucial for plant growth. In tobacco, the *NtAITR* gene negatively regulates drought tolerance through the ABA signaling pathway, and its knockout enhances drought resistance. The bZIP transcription factor *OsABF1* in rice participates in cold stress response by regulating trehalose synthesis. In *Camellia oleifera*, the *CoSPX-MFS3* gene enhances low-phosphorus adaptability by regulating phosphorus signaling pathways and organic acid accumulation. Genetic variation in the *TAL* effector gene of rice bacterial blight strain LA20 is the key factor enabling it to overcome rice resistance. In tomato, *SIWRKY80* enhances resistance to root-knot nematodes by activating SA/JA/ETH signaling and ROS metabolism. The *OsSPL42* gene in rice spotted-leaf mutant *spl42* affects disease resistance by regulating lignin biosynthesis and ROS balance. In potato, the *StPAM16-1* gene negatively regulates immune responses to common scab through interaction with the COP9 signalosome complex.

The authors of future studies should further elucidate the molecular mechanisms of key genes (*OsSPL42* and *StPAM16-1*) and their interactions with other pathways to refine our understanding of plant genetic regulatory networks. The application of gene-editing technologies such as CRISPR-Cas9 will enable precise modification of regulatory genes, facilitating the development of stress-resistant, high-yield, and high-quality crop varieties. Additionally, exploring the universality and potential of cross-species gene function validation (applying *OsCYP85A1* in woody plants) could broaden the scope of genetic improvement. Translating research findings into agricultural practice is crucial for breeding new crop varieties with enhanced resistance to diseases, drought, and low-phosphorus stress, ultimately improving crop yield and quality. Meanwhile, ecological balance must be prioritized to ensure the environmental safety of genetically improved crops and mitigate potential ecological risks. Strengthening interdisciplinary collaboration among the fields of molecular biology, genetics, ecology, and agronomy will drive comprehensive advancements in plant genetic regulation research. These efforts will provide scientific support for global food security and sustainable agriculture. In conclusion, continued in-depth research on plant genetic regulation will offer novel insights and tools to address agricultural challenges, contributing to the realization of sustainable agricultural development.
